# Key hydraulic traits control the dynamics of plant dehydration in four contrasting tree species during drought

**DOI:** 10.1093/treephys/tpad075

**Published:** 2023-06-15

**Authors:** Chris J Blackman, Lise-Marie Billon, Julien Cartailler, José M Torres-Ruiz, Hervé Cochard

**Affiliations:** ARC Centre of Excellence for Plant Success in Nature and Agriculture, School of Natural Sciences, University of Tasmania, Hobart 7001, Australia; Université Clermont-Auvergne, INRAE, PIAF, Clermont-Ferrand 63100, France; Université Clermont-Auvergne, INRAE, PIAF, Clermont-Ferrand 63100, France; Université Clermont-Auvergne, INRAE, PIAF, Clermont-Ferrand 63100, France; Université Clermont-Auvergne, INRAE, PIAF, Clermont-Ferrand 63100, France; Université Clermont-Auvergne, INRAE, PIAF, Clermont-Ferrand 63100, France

**Keywords:** cavitation, plant traits, SurEau

## Abstract

Trees are at risk of mortality during extreme drought, yet our understanding of the traits that govern the timing of drought-induced hydraulic failure remains limited. To address this, we tested SurEau, a trait-based soil–plant–atmosphere model designed to predict the dynamics of plant dehydration as represented by the changes in water potential against those observed in potted trees of four contrasting species (*Pinus halepensis Mill.*, *Populus nigra L.*, *Quercus ilex L.* and *Cedrus atlantica* (Endl.) Manetti ex Carriére) exposed to drought. SurEau was parameterized with a range of plant hydraulic and allometric traits, soil and climatic variables. We found a close correspondence between the predicted and observed plant water potential (in MPa) dynamics during the early phase drought, leading to stomatal closure, as well as during the latter phase of drought, leading to hydraulic failure in all four species. A global model’s sensitivity analysis revealed that, for a common plant size (leaf area) and soil volume, dehydration time from full hydration to stomatal closure (*T*_close_) was most strongly controlled by the leaf osmotic potential (*P*_i0_) and its influence on stomatal closure, in all four species, while the maximum stomatal conductance (*g*_smax_) also contributed to *T*_close_ in *Q. ilex* and *C. atlantica*. Dehydration times from stomatal closure to hydraulic failure (*T*_cav_) was most strongly controlled by *P*_i0_, the branch residual conductance (*g*_res_) and *Q*_10*a*_ sensitivity of *g*_res_ in the three evergreen species, while xylem embolism resistance (*P*_50_) was most influential in the deciduous species *P. nigra*. Our findings point to SurEau as a highly useful model for predicting changes in plant water status during drought and suggest that adjustments made in key hydraulic traits are potentially beneficial to delaying the onset of drought-induced hydraulic failure in trees.

## Introduction

Reports of drought-induced tree mortality are increasing in many forested regions of the world as temperatures rise and drought stress intensifies with global climate change (Allen et al. 2015, IPCC 2022). These reports are driving major research efforts into the mechanisms of tree mortality, and it is now widely recognized that widespread damage to the water transport system (hydraulic failure) is one of the leading causes of tree death during severe drought (Davis et al. 2002, Anderegg et al. 2016, Choat et al. 2018, Klein et al. 2022, McDowell et al. 2022). Hydraulic failure occurs when soil drying reaches critical levels and when the tension within the water-conducting xylem exceeds species thresholds, causing widespread cavitation and the formation of embolisms that occlude the water transport pathway (Tyree and Sperry 1989). Tree and woody shrub species exhibit a range of traits and response strategies that minimize their exposure to and ultimately delay the onset of embolism formation (Pivovaroff et al. 2016, Martin-StPaul et al. 2017, Blackman et al. 2019*a*). However, our ability to predict when individuals will sustain hydraulic failure during drought remains limited, largely because of a lack of knowledge of the specific traits and processes that contribute to determining plant dehydration times across diverse species.

The dynamics of plant dehydration during drought are broadly defined by the behaviour of stomata and their regulation of plant water loss. Under mild drought conditions, the stomata are open to facilitate photosynthetic gas exchange, with rates of plant and soil dehydration driven largely by the size and allometry of trees in combination with their intrinsic water transport capacity. As drought stress intensifies, stomata close to preserve the remaining water content in the soil, as well as within internal plant tissues, and prevent the onset of embolism formation (Buckley 2019, Creek et al. 2020). Nevertheless, plants continue to lose water through leaf cuticles and ‘leaky’ stomata (Schuster et al. 2017, Duursma et al. 2019, Machado et al. 2021) as well as through other tissues, including the bark (Wolfe 2020). These residual water losses slowly deplete internal water stores and water potentials continue to decrease, ultimately leading to hydraulic failure, complete tissue desiccation and plant death (Mantova et al. 2022). Taken together, the rates of water loss prior to and following stomatal closure influence the time it takes for a given tree to use up available soil water and reach a lethal level of dehydration. Nevertheless, in contrast to the considerable attention given to understanding plant resistance to embolism and stomatal regulation of plant water status during drought, our understanding of the traits and processes that shape the dynamics of plant dehydration and hydraulic dysfunction following stomatal closure remains limited (Choat et al. 2018).

The time needed for plants to reach critical levels of dehydration following stomatal closure depends on the interplay of several traits and processes that determine rates of water loss, total remaining water storage and the limits of drought tolerance in terms of xylem embolism resistance (Gleason et al. 2014, Blackman et al. 2016, Martin-StPaul et al. 2017, Cochard et al. 2021). Long dehydration times following stomatal closure should be maximal in species that exhibit low residual leaf (*g*_min_) or shoot (*g*_res_) conductance, large water storage reservoirs relative to leaf area, leaf shedding and high xylem cavitation resistance. However, trait trade-offs, for example, between the level of capacitive discharge of internal water reserves and xylem cavitation resistance (Pratt et al. 2007, Scholz et al. 2011), preclude certain combinations of traits. Thus, long survival times are achievable in species with contrasting traits and drought-response strategies (Pineda-Garcia et al. 2013, Pivovaroff et al. 2016), although some studies in realistic field settings have shown that the variation in survival times across species is largely explained by the resistance to embolism (Paddock et al. 2013, Venturas et al. 2016). Nevertheless, evidence from experimental and modelling studies clearly shows the importance of considering a range of traits when assessing drought mortality risk across diverse species (Wolfe 2017, Blackman et al. 2019*a*, 2019*b*, De Kauwe et al. 2020).

Process-based models aimed at simulating gas exchange and hydraulic responses under optimal and water-limiting conditions offer a promising way forward to better understand plant responses to drought (Mencuccini et al. 2019). Many of these describe how stomatal responses optimize carbon gain relative to the risks associated with hydraulic failure during drought (Sperry et al. 2017, Venturas et al. 2018). Yet, very few consider the water relations of plants under severe drought conditions at the very limit of their survival. To address this, the mechanistic soil–plant–atmosphere hydraulic model, SurEau, was recently developed to simulate the temporal dynamic of plant water status up to and beyond the point of stomatal closure, all the way through to levels of dehydration associated with hydraulic failure (Martin-StPaul et al. 2017, Cochard et al. 2021). In addition to the drivers of plant water use and stomatal responses to water stress, the model describes processes linked to the depletion of internal water storages and the formation of xylem embolisms. Martin-StPaul et al. (2017) were the first to present and use SurEau to test the hypotheses surrounding the role of coordinated stomatal closure and embolism resistance in determining drought mortality risk across diverse species. Since then, the model has been used to predict future increases in drought-induced tree mortality under a warming atmosphere (Brodribb et al. 2020) with rising CO_2_ (McDowell et al. 2022) and to simulate patterns of stomatal closure and cavitation during drought (Lamarque et al. 2020, López et al. 2021). Importantly, SurEau enables predictions to be made of the time to hydraulic failure (THF), that is, the time it takes for trees (and crops) to use up available soil water and dehydrate to the water potentials associated with critical losses of hydraulic conductivity due to embolism (Lemaire et al. 2021). However, the model remains to be directly validated against changes in the plant water status in tree species exposed to experimental drought.

In this study, we provide the first direct experimental validation of the dynamics of dehydration in multi-year-old trees growing in large containers, as predicted using the model SurEau. We parameterized SurEau with a range of hydraulic, allometric and drought-response traits, either measured in potted trees or sourced from previous studies, of two angiosperm and two conifer species known to exhibit contrasting water-use and drought-adaptation strategies. The predicted dynamic of plant water potential was tested against those observed in experimental plants during an imposed extreme drought by withholding water. We also applied a global sensitivity analysis, with the aim of elucidating the relative contribution of different traits to the predicted time for plants to reach critical levels of drought stress prior to and following stomatal closure. We expected that, when normalized by canopy area, pot size and climate during drought, dehydration times from full hydration to water potentials at stomatal closure would be driven most strongly by ‘water-use’ traits, including maximum stomatal conductance (*g*_smax_) and stomatal closure. By contrast, we expected that dehydration times from water potentials at stomatal closure through to the point of hydraulic failure would be driven most strongly by a combination of ‘water-loss’ traits, such as branch residual conductance (*g*_res_), and drought-tolerance traits such as xylem cavitation resistance (*P*_50_).

## Materials and methods

### Species and experimental design

A group of four tree species, consisting of two angiosperms (*Populus nigra* L. and *Quercus ilex* L.) and two conifers (*Cedrus atlantica* (Endl.) Manetti ex Carrière and *Pinus halepensis* Mill.), were selected for their contrasting water-use and drought-tolerance traits. *Populus nigra* is a fast-growing broad-leaf deciduous species restricted to low-lying areas with high moisture availability. *Quercus ilex* is an evergreen species native to the Mediterranean region, where its distribution overlaps with *P. halepensis*. *Cedrus atlantica* is native to the Atlas Mountains in northwest Africa. One-year-old saplings of *P. nigra* and 2- to 3-year-old saplings of the remaining three species, all propagated as cuttings, were sourced from a local nursery and were planted in large 100-l (56 cm high × 52 cm wide; *P. halepensis* and *Q. ilex*) or 80-l (50 cm high × 46 cm wide; *C. atlantica* and *P. nigra*) pots located at the INRAE site in Crouel, Clermont-Ferrand, central France (45.7772° N, 3.0870° E) at an elevation of 350 m. The trees were grown under well-watered (WW) conditions over multiple years, with plant age at the start of the experiment ranging from 3 years for the fast-growing *P. nigra* and from 5 to 6 years for the remaining three species. The trees were grown outside, except for the winter months of each year, when all individuals were moved to nearby glasshouses to avoid frost damage. The medium used to grow the plants was a sandy clay soil with a relative water content at field capacity of 37% and residual water content of 9%.

In May 2019 (Northern Hemisphere Spring), 18 potted individuals of each species were moved to a rain exclusion facility at the INRAE site and were placed on high-capacity (300 kg, 50 g resolution) balances (PW12CC3, HBM, Darmstadt, Germany), programmed to record pot mass (g) every 5 min. The exposed soil of each pot was covered with cling film to minimize water evaporation. Rain was excluded from the plants by a large retractable open-ended trapezoid transparent shelter. A rain gauge positioned on a nearby roof triggered the shelter to close (i.e., the shelter moved over top of the plants) during periods of rain and reopen when the rain stopped. A weather station was installed close to the shelter, providing hourly climatic data for air temperature, humidity, photosynthetically active radiation and wind speed.

**Figure 1:**
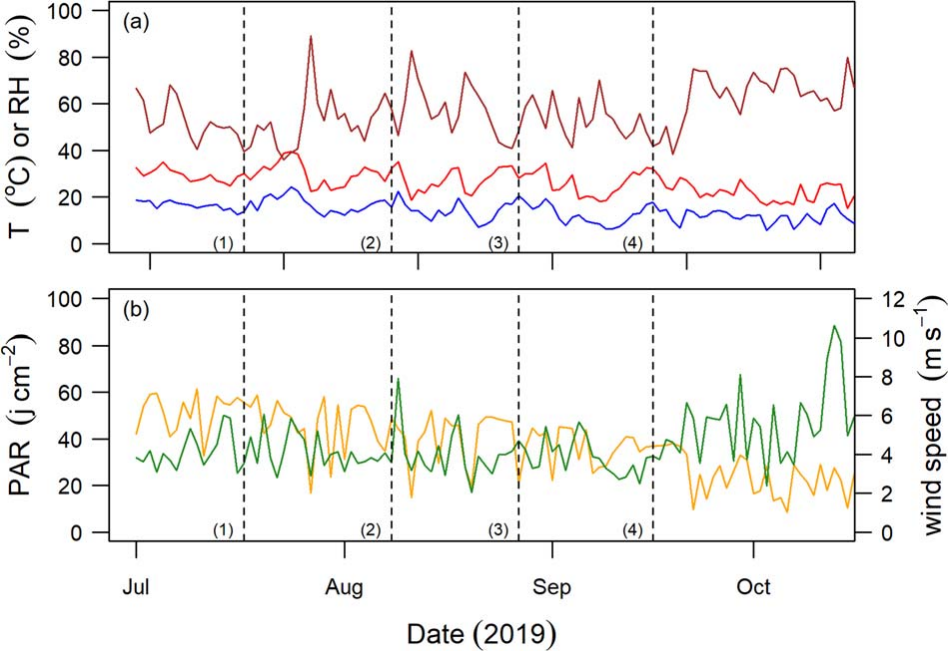
Climate variables recorded on-site during the dry-down experiment. Plot (a) shows minimum (blue) and maximum (red) daily temperature and mean daily relative humidity (brown). Plot (b) shows mean daily photosynthetic active radiation (yellow) and wind speed (green). Numbered dashed lines indicate the start date of each species dry-down treatment: *P. halepensis* (1), *P. nigra* (2), *Q. ilex* (3) and *C. atlantica* (4).

Twelve plants per species were assigned to a water-stressed (WS) treatment, while six plants per species were assigned as WW controls. All plants were initially maintained under WW conditions, with water being added to the soil via an automated irrigation system programmed to maintain the soil water content at saturation. Drought was applied to the WS plants by disconnecting the irrigation and withholding water. For the purposes of improved experimental management, the beginning of each species dry-down treatment was staggered, with *P. halepensis* commencing on 10 July 2019, *P. nigra* commencing on 2 August 2019, *Q. ilex* commencing on 11 August 2019 and *C. atlantica* commencing on 9 September 2019 (see Figure 1).

### Allometry

Maximum canopy leaf area (LA; m^2^) was estimated for individual trees of each species from the allometric relationships developed between LA and stem volume (m^3^). Six to eight side-branches differing in size and position were cut from the main stem from each of five to six WW plants per species. Stem volume was calculated from the measurements of branch length and basal diameter, assuming the shape of a cylinder, while LA was calculated by multiplying the branch leaf dry weight (g) by species mean specific leaf area (SLA; m^2^ g^−1^). The SLA was determined from the relationship between projected area (m^2^) and dry weight (g) of 5–10 representative leaves sampled from 5 WW trees per species. For individuals of each species, the total canopy LA was calculated by measuring the volume (length and basal diameter) of all branches arising from the main stem and by solving the linear regression equation fitted to the relationship between LA and stem volume across the sampled branch sizes. The height and basal diameter of the main stem of each individual were also measured and its volume was calculated. See Table 1 for species allometric values.

**Table 1:** Plant allometric, gas exchange and hydraulic trait means used to parameterize the model SurEau for predicting the dynamics of plant desiccation during drought for the four study species. Also included are the response variables, *Ψ*_min_ and PLC, recorded for each species at the end of the experimental drought. Note that allometric values represent species means, yet model simulations were run using individual-level data.

	Units	*n*	*Pin_hale*	*Pop_nigr*	*Que_ilex*	*Ced_atla*
Canopy leaf area	m^2^	18	3.64	1.94	1.77	1.24
Height	m	18	2.08	1.24	1.49	1.14
Basal diam.	cm	18	5.7	1.9	3.6	3.8
*P*_50_	MPa		−4.67^1^	−2.2^1^	−7.13^1^	−5.14^1^
Slope	%MPa^−1^		78	100	23	69
TLP	MPa	5	−2.15	−2.07	−2.83	−3.14
*P*_i0_	MPa	5	−1.26	−1.52	−1.89	−2.14
$\varepsilon $	MPa	5	9.27	15.9	16.2	13.0
*g*_smax_	mmol s^−1^ m^−2^		60	220	100	120
*P*_gs88_	MPa	6	−2.96	−1.14	−4.04	−2.57
*g*_res_	mmol s^−1^ m^−2^	5	0.58	4.5	1.19	1.21
*T*_p_	°C	5	42.1	35.0	43.2	41.3
*Q*_10a_		5	1.13	1.2	1.06	1.22
*Q*_10b_		5	2.99	4.8	1.52	1.83
*Ψ*_min_ (WS trees)	MPa	12	−5.25	−2.54	−8.61	−3.46
PLC (WS trees)	%	6–8	60.8	82.5	–	22.7

Notes: *Pin_hale* = *Pinus halipensis*; *Pop_nigr* = *P. nigra*; *Que_ilex* = *Q. ilex*; *Ced_atla* = *C. atlantica*; *P*_50_ = 50% loss in xylem hydraulic conductivity; *P*_i0_ = osmotic potential at full turgor; $\varepsilon $ = modulus of elasticity; *g*_smax_ = maximum stomatal conductance; *P*_gs88_ = 88% stomatal closure; *g*_res_ = minimum branch conductance; *T*_p_ = phase transition temperature; *Q*_10a_ and *Q*_10b_ = *Q*_10_ of the relationship between *g*_res_ and the temperature below and above *T*_p_; *Ψ*_min_ = minimum water potential during drought; PLC = percentage loss in conductivity measured in stems immediately after peak drought stress.

^1^Indicates *P*_50_ values sourced from previous studies: *Pin_hale* (Cochard 2006); *Pop_nigr* (H. Cochard, unpublished); *Que_ilex* (Lobo et al. 2018); *Ced_atla* (Cochard 2006).

### Dry-down treatment

For each species, the WS treatment was commenced by stopping irrigation and by allowing trees to use up available soil water and dehydrate to target water potentials associated with at least 50% loss of stem hydraulic conductivity (*P*_50_). These water potentials were drawn from the reference hydraulic vulnerability curves sourced from previous studies (*P*_50_; −4.67 MPa in *P. halepensis*, −2.2 MPa in *P. nigra*, −7.13 MPa in *Q. ilex* and −5.14 MPa in *C. atlantica*; see further details below). All WS trees reached their respective target water potential, with the exception of *C. atlantica*, which were dried down late in the season and only reached water potentials of ca −3.5 MPa before cooler and more humid weather set in. Dead leaves were collected daily for WS individuals of *P. nigra* following a strong leaf shedding response during drought. Leaf death was minimal (<10% of total initial canopy area) in the three evergreen species.

Once individual trees had reached their target water potential, pots were rewatered to field capacity by repeat watering until soils became saturated. During the days and weeks after rewatering, all individuals showed signs of recovery as indicated by a ‘re-greening’ of canopy foliage. Indeed, no trees were killed outright by the drought, though evidence of branch dieback was apparent in *P. nigra*. Levels of hydraulic dysfunction due to embolism were confirmed in six to eight individual WS trees, as well as the WW controls, by sampling branches shortly after rewatering, and using an X-ray microtomograph (Nanotom 180 XS, GE, Wunstorf, Germany) to measure embolism accumulation expressed as a percentage loss of xylem function (see Torres-Ruiz et al. 2015, 2016 for details of the micro-CT technique). In brief, a single ~1-cm diameter side-branch ~50 cm in length was cut in the air from each tree in the early morning and was immediately wrapped in a moist paper towel placed inside humidified plastic bags. For *Q. ilex*, which has long vessels, branches or whole shoots >1 m in length were sampled. In the laboratory, individual branches were immersed in water and were gradually cut back carefully from each end to a length of ~10 cm. Samples were immediately coated in paraffin wax before being cold-room-stored and subsequently scanned. After this first scan, the samples were cut with a clean razor blade just above the scanned area and were scanned again. Thus, xylem conduits that were functional (i.e., full of water) during the first scan will embolize because the xylem sap is under negative pressure. The percentage loss of xylem function was determined by evaluating the proportion of functional conduits before and after this second cut. Despite careful handling, the micro-CT images of *Q. ilex* stems collected from both WS and WW trees were significantly embolized (>80%) as an artefact of branch cutting and preparation (Torres-Ruiz et al. 2015), and thus we were not able to assess the levels of embolism due to drought. Some WW *P. nigra* individuals were also embolized (up to 50%), possibly due to measurement artefacts, but were significantly less embolized than droughted plants of the same species (>80%). Supporting Information (Figure S1 available as Supplementary data at *Tree Physiology* Online) shows micro-CT images of a representative sample collected from WW and WS trees of each of the four species, shortly after rewatering from peak drought.

### Drought-response measurements

Predawn and midday xylem water potentials (MPa) were measured in WW and WS trees every 2–5 days using a Scholander-type pressure chamber (PMS Instrument Company, Albany, OR, USA), though midday water potentials were measured less frequently in *P. nigra* and *C. atlantica* to conserve available LA material. The evening prior to each measurement date, an individual leaf from the middle to upper canopy of each tree, or small shoot in the case of *P. halepensis* and *C. atlantica*, was covered by an aluminium-lined plastic sleeve to prevent leaf transpiration and to ensure that measurements represented xylem water potentials. Over the course of each species drought experiment, water potentials in WS plants generally showed a steady decrease, with relatively low variability among individuals even at dehydration levels approaching peak stress in three of the four species. The exception to this was *P. nigra* where leaf shedding in response to drought may have caused water potentials to vary as WS plants approached peak stress. Nevertheless, while noting that water potentials can be variable within individual canopies under severe drought, we were careful in targeting leaves from within the same canopy position throughout the experiment.

We calculated the daily midday canopy transpiration in WW and WS trees by normalizing the mass loss of water, which was captured via the balances, by canopy LA over the course of 2 h around midday. In WS trees, the water potential at stomatal closure (*P*_gs88_) was identified by fitting a ‘Weibull’ function to the midday *E* versus water potential data across trees of each species, respectively (Figure S2 available as Supplementary data at *Tree Physiology* Online).

### Hydraulic traits

For each species, parameters linked to xylem hydraulic vulnerability to drought-induced embolism were drawn from a reference vulnerability curve, sourced either from previous studies or from previous unpublished data (*P. halepensis*, Cochard 2006; *P. nigra*, H. Cochard, unpublished; *Q. ilex*, Lobo et al. 2018; and *C. atlantica*, Cochard 2006). In each case, the vulnerability data were generated using the Cavitron technique (Cochard et al. 2005) on material collected from trees growing locally under climate conditions similar to that of our experimental trees. The hydraulic vulnerability of leaves and stems was assumed to be the same within species. While this may be true for some woody species (e.g., Skelton et al. 2018), others show strong vulnerability segmentation (e.g., Zhu et al. 2016).

Pressure–volume curves were generated for each species using bench dehydration (Tyree and Hammel 1972). A single leaf of *P. nigra*, or small shoot of *P. halepensis*, *Q. ilex* and *C. atlantica*, was sampled from four to six individuals, respectively. The relationship between the relative water content and water potential measured during bench dehydration was used to determine the turgor loss point (TLP, MPa), solute potential at full turgor (MPa) and leaf modulus of elasticity ($\varepsilon $, MPa).

Minimum residual conductance (*g*_res_, mmol m^−2^ s^−1^) and its phase transition temperature (*T*_p_) was determined using six to eight small branches, per species, dried-down inside a climate-controlled Drought-box (Billon et al. 2020). Branches, 40- to 50-cm long, were hydrated overnight, recut in air and the cut end was sealed with melted paraffin wax. Each branch was measured for length (mm), basal diameter (mm) and saturated mass (g) before being suspended on one of eight strain gauges inside the Drought-box. Climate conditions were initially set to 30 °C and 40% relative humidity and the branches were allowed to dehydrate, with changes in mass representing the branch water loss. Stomatal closure occurred after a period of between 2 and 6 h, depending on species. After 24 h inside the Drought-box, branches were removed, their leaves and stems were separated and were oven-dried at 70 °C. The *g*_res_ was determined from the water loss data beyond the point of stomatal closure. To do this, rates of water loss were normalized by the vapour pressure deficit (VPD; KPa) recorded inside the Drought-box and the surface area of both the main stem (as represented by a cylinder) and double-sided LA for the two angiosperms and projected LA for the two conifer species. The LA was calculated by multiplying the total leaf dry weight of each branch by species mean SLA.

The phase transition temperature (*T*_p_, °C), which defines the temperature point at which *g*_res_ markedly increases across a range of temperatures, was determined on a separate set of branches sampled from the WW plants of each species and dried down in the Drought-box (Billon et al. 2020). In these experiments, branches were dried down under six stepwise increases in temperature (30, 35, 40, 45, 50 and 55 °C), with concurrent decreases in relative humidity (40, 30, 23, 18, 14 and 11%) to maintain the water concentration of air at 10.6 g kg^−1^. The *g*_res_ was calculated at each temperature step as described above. From these data, we calculated the *T*_p_ for each species using an Arrhenius plot as described in Bueno et al. (2019), while *Q*_10a_ and *Q*_10b_ were calculated as the *Q*_10_ of the relationship between *g*_res_ and the temperature below and above the *T*_p_ value, respectively.

### Modelled dynamics of plant dehydration

SurEau is a mechanistic soil–plant–atmosphere hydraulic model which predicts plant responses under simulations of water stress. It works by segmenting the soil–plant–atmosphere into compartments, with water fluxes between segments determined by the gradients of water potential. In the model, the hydraulic properties of leaf symplastic and stem apoplastic compartments are calibrated from the pressure–volume curves and xylem vulnerability curves, respectively. In doing so, SurEau accounts for both the buffering effect of symplastic capacitance on water fluxes under well-hydrated conditions and the early onset of drought and the contribution of apoplastic water during more severe conditions once xylem cavitation begins to occur. A detailed description of the model and its function, including equation derivations and code, is provided by Cochard et al. (2021).

We modelled the dynamics of plant dehydration, as represented by changes in the plant water potential (in MPa). To do this, we parameterized SurEau with plant (hydraulic and allometric) traits and the specific climatic conditions associated with each of the four species during their respective dry-down experiments (see Table S1 available as Supplementary data at *Tree Physiology* Online). Drought simulations were performed on all WW and WS individuals of each species, with each simulation starting at soil saturation and ending at hydraulic failure of the plant, defined as the water potential associated with 99% loss of leaf hydraulic conductivity. Model input consisted of variables specific to each species drawn either from measurements on experimental plants in the current study or from measurements on the same species reported in previous studies. Measurements made on experimental plants included species means for pressure–volume traits, branch *g_res_* and *T*_p_ and allometric traits such as plant height, basal diameter and leaf area. These allometric traits were used to generate a ‘fractal’ tree with associated trunk, branch and root dimensions (length and area) and conductances (see Cochard et al. 2021). Maximum stomatal conductance (*g*_smax_) was determined for each species by tuning *g*_s_ values to match midday transpiration rates measured for WW plants, based on the equation *E* = *g*_s_^*^VPD/*P*, where *P* is the atmospheric pressure. Hydraulic vulnerability curves were sourced from the literature (see Table 1).

For model simulations, stomates were set to respond to the changes in leaf turgor, as computed using leaf osmotic potential and the modulus of elasticity derived from each species pressure–volume curves. Minimum conductance from the bark (*g*_bark_) was assumed to be double that of the leaves and shoots (*g*_res_) following recent evidence across diverse species in the field (Loram-Lourenço et al. 2022). A leaf shedding function fitted to experimental data was used for *P. nigra*, whereby the percentage of shed leaves (PLF) followed a sigmoidal response to leaf water potential: PLF = 100/(1 + exp(4^*^(*P*_leaf_ + 1.8))). Climate input variables were drawn from the hourly climate data measured on site during each species dry-down experiment. Soil input parameters used to calculate field capacity, etc, were constant across species. Input variables related to the length of roots was calculated using a fractal representation of tree allometry (see Cochard et al. 2021). Table 1 provides a summary of selected trait means for each species, while Table S1, available as Supplementary data at *Tree Physiology* Online, provides a full list of model input variables.

### Sensitivity analysis

A global model’s sensitivity analysis using the R package ‘sensobol’ (Sobol 2001) was performed to elucidate the plant traits that contribute to variation in THF, as predicted by SurEau. This analysis targeted the contribution of a range of 34 representative plant allometric and hydraulic traits (see Tables S1 and S2 and Figure S3 available as Supplementary data at *Tree Physiology* Online) and did not include the climatic variables and soil characteristics, such as soil depth or total leaf area, which were assumed to be equal across the four species. Two different phases were deemed to influence the dynamics of plant dehydration during drought, depending on whether the leaf xylem water potential occurred above or below the stomatal closure point. Thus, two time-based metrics were used in the sensitivity analysis to explore the relative contribution of input variables to plant dehydration times: (i) from full hydration to water potentials at stomatal closure (*T*_close_) and (ii) from stomatal closure to water potentials at hydraulic failure, defined here as 99% loss of hydraulic conductivity due to embolism (*T*_cav_). An additional metric of the overall time from full hydration to the point of hydraulic failure was also defined (THF). We conducted the sensitivity analysis by generating pseudo-random simulations of each species (40,000 simulations in total for each species), with trait values randomly sampled from a ±10% range of the trait mean or value used to model the dynamics of plant dehydration (see above). We report ‘Sobol’s total order indices’ that quantify the relative contribution of each of the 34 parameters (i.e., hydraulic traits) to the variance of THF (Figure S3 available as Supplementary data at *Tree Physiology* Online). Simulations were run with plant size, LA and soil volume set constant across species, while the daily climate inputs were set constant according to the conditions typical of a summer’s day.

## Results

### Modelled versus observed plant dehydration

**Figure 2:**
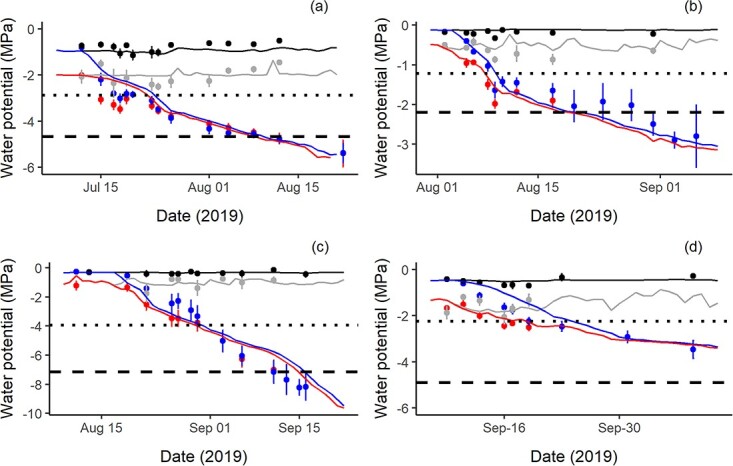
Plots showing strong correspondence between observed and modelled water potential dynamics during drought in each of the four study species: (a) *P. halepensis*, (b) *P. nigra*, (c) *Q. ilex* and (d) *C. atlantica*. In each plot, the colouring and symbol type indicate the mean (±SE) observed (circles) and mean modelled (lines) predawn and midday water potentials for WS (blue and red lines, respectively) and control (WW; black and grey lines, respectively) plants. In each plot, the horizontal lines indicate the water potential at stomatal closure (*P*_gs88_; dotted line) and 50% loss of hydraulic conductivity (*P*_50_; dashed line).

We observed a strong agreement between the predicted (model output) and observed dynamics of plant dehydration during the different phases of drought in each of the four study species, respectively (Figure 2). In all species, following the cessation of water input, the predicted and observed predawn and midday xylem water potentials declined rapidly and converged at, or shortly after, the point of stomatal closure. During the latter phase of drought, from the point of stomatal closure though to hydraulic failure, modelled rates of water potential slowed and closely matched those observed in experimental plants of each species. However, a slight decoupling was observed for the deciduous species *P. nigra*, which showed a strong leaf shedding response in experimental trees, which acted to slow the observed rates of water potential decline.

Except for *C. atlantica*, the minimum water potential at peak drought for individual WS trees corresponded to or exceeded each species target water potential at the reference stem *P*_50_ (Table 1). For each species, these minimum water potentials corresponded to an average level of percentage loss of conductivity (PLC) due to the embolism of 61% in *P. halepensis*, 83% in *P. nigra* and 23% in *C. atlantica* (Table 1), with PLC values recorded in WS individuals showing a good correspondence with those predicted at similar water potentials from each species reference vulnerability curve (Figure S4 available as Supplementary data at *Tree Physiology* Online). The PLC values in WW trees remained <20% in *P. halepensis* and *C. atlantica* but reached up to 50% in *P. nigra*, possibly due to measurement artefacts.

We also observed a strong correspondence between the predicted and observed dynamics in whole-tree transpiration as represented by the changes in relative extractable soil water content during drought (Figure S5 available as Supplementary data at *Tree Physiology* Online).

### Species rank in THF

**Figure 3:**
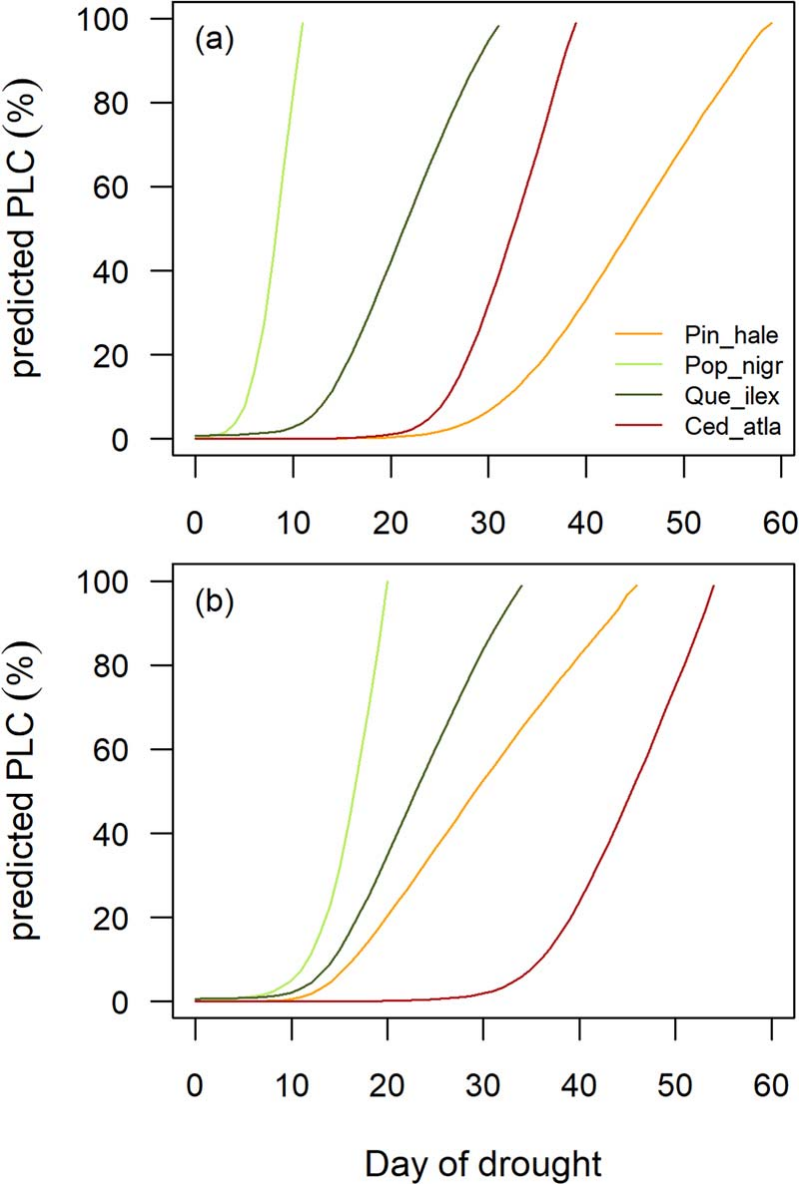
Modelled dynamics of percentage loss of conductivity (PLC) due to embolism during a simulated drought in the four study species (orange = *P. halepensis*; light green = *P. nigra*; dark green = *Q. ilex*; red = *C. atlantica*). In plot (a), plants of each species were standardized by LA and pot size and dried down under the same climatic conditions. In plot (b), LA was allowed to vary according to species differences recorded in experimental plants.

When standardized for LA and soil volume, and when dried down under the same climatic conditions, plants of the four study species varied in their predicted THF, with longer THF predicted for the two conifer species compared with the two angiosperms (Figure 3a). Overall, the shortest THF was predicted for plants of the deciduous species *P. nigra*, while the longest was predicted for plants of *P. halepensis*. When using non-standardized data and allowing for the differences in plant size and LA recorded in the experimental plants (but maintaining common soil and climatic variables), species rank was slightly altered, with the longest THF predicted for *C. atlantica* (Figure 3b). Among the four study species, the predicted THF using standardized data (Figure 3a) was weakly correlated with branch residual conductance (*g*_res_) but was related neither to the water potential at stomatal closure (*P*_gs88_) and hydraulic vulnerability (*P*_50_) nor to the species hydraulic safety margin, defined as the difference between *P*_gs88_ and *P*_50_ (Figure S6 available as Supplementary data at *Tree Physiology* Online). When allowing for the differences in plant size and leaf area, no significant relationships were observed between the predicted THF and individual hydraulic traits.

### Traits that contribute most to THF

**Figure 4:**
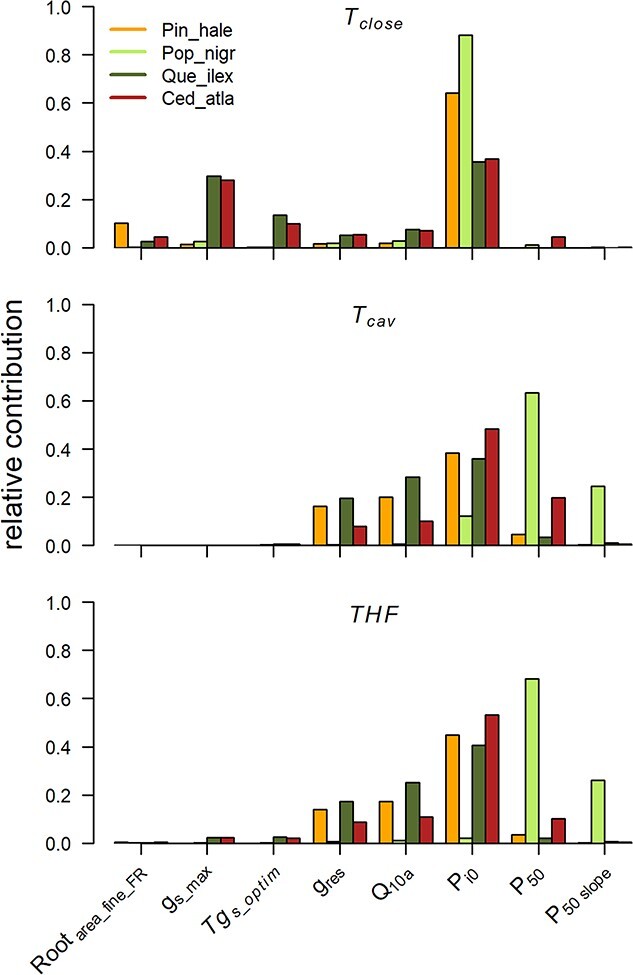
A global sensitivity analysis, highlighting the relative contribution (i.e., their Sobol’s total order indices) of key allometric and hydraulic traits to determining each species predicted dehydration time from full hydration to stomatal closure (*T*_close_), the subsequent THF associated with 99% loss of conductivity (*T*_cav_) and the overall time from full hydration to hydraulic failure (THF). These traits were included in the analysis of 34 traits and were selected on the basis that they contributed >10% to the total variance of at least one dehydration time for any one species. Traits ending with ‘FR’ were calculated via a representative ‘fractal tree’ (see Cochard et al. 2021). Colours represent the four species in the study: *Pin_hale* = *Pinus halepnsis* (orange); *Pop_nigr* = *P. nigra* (light green); *Que_ilex* = *Q. ilex* (dark green); *Ced_atla* = *C. atlantica* (red). See Table 1 and Table S1, available as Supplementary data at *Tree Physiology* Online, for full trait details, and see Table S3 and Figure S3, available as Supplementary data at *Tree Physiology* Online, for the sensitivity analysis output of all 34 traits for each species.

Global sensitivity analysis revealed that, for each species, the predicted time to stomatal closure (*T*_close_) was most strongly controlled by water-use traits, including leaf osmotic potential (*P*_i0_) and maximum stomatal conductance (*g*_smax_) in *Q. ilex* and *C. atlantica* (Figure 4 and Figure S3 available as Supplementary data at *Tree Physiology* Online). For the three evergreen species, the THF following stomatal closure (*T*_cav_), as well as overall THF, was largely determined by *P*_i0_, branch residual conductance (*g*_res_) and the *Q*_10a_ sensitivity of *g*_res_. By contrast, the predicted THF for the one deciduous species *P. nigra* was most strongly controlled by xylem embolism resistance (*P*_50_).

## Discussion

### Predicting tree mortality risk during drought

In this study, we explicitly tested the mechanistic soil–plant–atmosphere model, SurEau, and its ability to predict changes in the water status in experimental plants exposed to drought. For each species, including three evergreen and one deciduous species, we found an overall strong correspondence between the predicted and observed dynamics of plant water potential during the early phase of dry-down, leading to stomatal closure, as well as during the latter phase of dry-down, leading to hydraulic failure. These findings suggest that SurEau can be used to predict the dynamics of plant dehydration as well as the timing of key physiological responses during drought, including stomatal closure and the dynamics of embolism formation. Given that embolism-induced hydraulic failure is the leading mechanism of tree death when exposed to extreme drought (Adams et al. 2017, Nolan et al. 2021), this predictive capacity may greatly improve our ability to assess the species mortality risk under future climates.

Our findings build on recent work highlighting the importance of incorporating hydraulic traits into soil–plant–atmosphere models of tree mortality risk during drought (Sperry and Love 2015, Martin-StPaul et al. 2017, Mencuccini et al. 2019, De Kauwe et al. 2020, Cochard et al. 2021), including a recent study that uses a newly developed version of SurEau (SurEau-Ecos) to predict tree mortality at stand and regional scales (Ruffault et al. 2022). The SurEau is parameterized with traits that are commonly measured in plant growth and eco-physiological studies, including plant allometry, leaf gas exchange, pressure–volume dynamics, leaf and shoot residual conductance and hydraulic vulnerability. Unlike the many studies on plant responses to drought in seedlings, we droughted multi-year-old trees with structural and hydraulic traits, which more closely resemble trees of the same species growing in the field. This was evidenced by similar levels of embolism recorded in individuals at peak drought and those predicted from the reference vulnerability curves for three of the four study species. Furthermore, these results provide a level of support for using thresholds of drought-induced embolism in our modelled THF, which were derived from these vulnerability curves. However, we note that caution should be applied here considering that cavitation vulnerability can change in response to growing conditions in some species, especially broad-leaved angiosperms such as *Populus* (Lemaire et al. 2021).

Despite using large pots designed to buffer the shock of experimental drought stress, we also acknowledge that key traits relating to species differences in rooting characteristics, especially rooting depth and root residual conductance, were absent from the experiment, which limits how well our results translate to trees in the field. Indeed, even though *C. atlantica* was predicted to exhibit longer THF than the other three experimental species, this result is not necessarily transferable to the field without knowledge of species rooting depth and native soil parameters. Characterizing these variables across the landscape alongside factors related to tree size and stand density remains a significant challenge for understanding and ultimately modelling the dynamics of tree dehydration during drought (Fensham and Fairfax 2007, Nardini et al. 2016, Jacobsen and Pratt 2018, Ripullone et al. 2020, Chitra-Tarak et al. 2021, Pivovaroff et al. 2021, Britton et al. 2022).

We observed a strong correspondence in the dynamics of plant dehydration, leading to hydraulic failure in the three evergreen species, and a slightly weaker correspondence in the deciduous species *P. nigra*, which showed a strong leaf shedding response over the course of a few days during drought. Leaf shedding is well known as an adaptive response to drought, especially in tropical dry forests (Mendez-Alonzo et al. 2012, Wolfe et al. 2016, Ribeiro et al. 2021), and it is typically associated with species that exhibit hydraulic vulnerability segmentation (Tyree and Ewers 1991, Tyree et al. 1993). In such species, the shedding of vulnerable leaves during drought is thought to minimize hydraulic dysfunction in carbon-expensive stems. In the current study, we included a leaf shedding function in the model that described changes in the canopy LA with decreasing water potential in *P. nigra* trees, yet we still observed a slight decoupling of modelled versus observed dehydration dynamics around the time that leaves were being shed. This suggests that the function used did not adequately capture the influence of leaf shedding on the plant water potential in experimental trees. Future modelling work should aim to quantify leaf shedding responses during drought and test for the potential differences between leaf and stem xylem embolism resistance as a function of hydraulic vulnerability segmentation (but, see Lemaire et al. 2021).

### Traits that contribute to THF

Global sensitivity analysis revealed that a relatively consistent set of input parameters controlled most of the variation in predicted THF. For each species, the time for plants to reach water potentials at stomatal closure (*T*_close_) was driven largely by ‘water-use’ and drought-response traits, especially the leaf osmotic potential at full turgor (*P*_i0_) and maximum stomatal conductance (*g*_smax_) in two species, both of which influence rates of plant transpiration and draw-down of available soil water. Leaf osmotic potential, in particular, is widely recognized as a key drought-tolerance trait across global species as a result of its contribution to determining turgor loss and stomatal closure (Bartlett et al. 2012). Furthermore, it is reported to be highly plastic in plants subject to variable water availability (Bartlett et al. 2014, Mitchell and O'Grady 2015). Thus, levels of osmotic adjustment will likely have a major influence on how quickly trees and other plants use up available soil water and close their stomata during drought. This includes a shortening of the overall THF in plants that make adjustments in *P*_i0_ in response to growth under soil water deficit, which acts to delay turgor loss and stomatal closure, although this effect is negated in plants that also increase their xylem cavitation resistance (Lemaire et al. 2021).

The time for plants to reach critical levels of drought stress associated with hydraulic failure following stomatal closure (*T*_cav_) was controlled most strongly by a combination of ‘water-loss’ traits, including *P*_i0_, residual branch conductance (*g*_res_) and the *Q*_10a_ sensitivity of *g*_res_ to increasing temperature in the three evergreen species, *P. halepensis*, *Q. ilex* and *C. atlantica*. By contrast, *T*_cav_ was most strongly controlled by the traits linked to xylem embolism resistance, especially xylem *P*_50_, in the deciduous species *P. nigra*. Among all of these traits, the contribution of *Q*_10*a*_ and its influence on minimum conductance under increasing temperatures highlight the need to consider traits that influence plant dehydration times under variable climatic conditions, which is important considering that trees are at a greater risk of mortality during hot-drought conditions (Allen et al. 2015, Hammond et al. 2022).

These sets of traits that controlled *T*_cav_ also influenced the overall THF, from full hydration through to 99% loss of hydraulic conductivity, highlighting the importance of measuring ‘water-loss’ and drought-tolerance traits when assessing the species mortality risk during drought (Brodribb et al. 2020). Nevertheless, it is also important to note that, when standardized for plant size, soil volume and climate, the rank order of modelled THF among our four study species was not strongly correlated with any single drought-response or drought-tolerance trait, including hydraulic vulnerability to embolism. This re-emphasizes the need to consider a range of traits in understanding the different ways in which plant species may respond to drought and delay the onset of hydraulic failure (Pivovaroff et al. 2016, Blackman et al. 2019*a*).

The specific traits or set of traits controlling THF varied between the evergreen and deciduous species in this study. The set of traits controlling THF was largely consistent across the three evergreen species, suggesting a similarity in the relative sensitivity of traits for delaying the onset of hydraulic failure. It also suggests that these evergreens can potentially minimize the risk of hydraulic failure during drought under future climates by adjusting more than one trait. By contrast, adjustments in the xylem embolism resistance were predicted to provide the most benefit for delaying the onset of hydraulic failure in the deciduous species *P. nigra*. Whether plants make these adjustments under future climates will depend not only on their ‘cost’ to different aspects of plant function, for example, the potential cost of increased embolism resistance in terms of reduced hydraulic efficiency (Holtta et al. 2011, Koçillari et al. 2021), but also on a species adaptive capacity to alter their phenotype in response to environmental change (Aspinwall et al. 2015). For example, lower levels of residual leaf conductance in plants grown under soil water deficit and/or elevated temperatures have been reported in some (Duursma et al. 2019) but not in other species (Bueno et al. 2020). Meanwhile, the capacity to adjust the xylem embolism resistance is low in the species of pine (Lamy et al. 2014, Lopez et al. 2016) but may be higher in cool temperate angiosperms, especially deciduous species (Schuldt et al. 2016, Torres-Ruiz et al. 2019, Lemaire et al. 2021). We acknowledge that LA was not included in our sensitivity analysis but that it has been shown to be highly plastic in response to growth under conditions of variable water availability and is predicted to have a strong influence on plant dehydration times in individuals (Lemaire et al. 2021) and forest systems (Ruffault et al. 2023). Furthermore, dehydration times are also likely to be influenced via changes in resource allocation such as the sapwood to LA ratio (Mencuccini and Bonosi 2001) and root growth (Chitra-Tarak et al. 2021) in response to changes in water availability.

## Conclusions

Hydraulic failure is, arguably, the leading cause of tree death during extreme drought events. Given that forest trees in numerous parts of the world are facing an increased drought severity as global temperatures continue to rise, there is an urgent need to develop process-based models that can predict patterns of dehydration leading to mortality during drought and heatwave conditions. In this paper, we show how SurEau, a soil–plant–atmosphere model, can predict the temporal changes in plant water status under variable climatic conditions and thus the timing of the onset of critical levels of drought stress associated with hydraulic failure. We also show that a few key hydraulic traits control longer dehydration times across species. Understanding how these traits vary within and across diverse species, as well as their responsiveness to changes in climatic conditions, is a priority for future research. We acknowledge that a number of challenges remain for process-based models such as SurEau to adequately capture the variation in traits that contribute to determining plant dehydration times. These include accurately measuring LA and rooting depth in mature trees in the field and capturing the drought-response processes linked to root growth and leaf shedding as well as interactions with neighbours in mixed forest systems.

## Supplementary Material

Supporting_Information_Fig_S1_tpad075

Supporting_Information_Fig_S2_tpad075

Supporting_Information_Fig_S3_tpad075

Supporting_Information_Fig_S4_tpad075

Supporting_Information_Fig_S5_tpad075

Supporting_Information_Fig_S6_tpad075

Supportin_Information_Tables_1_tpad075

## Data Availability

Data are available upon request to the authors.

## References

[ref1] Adams HD, Zeppel MJB, Anderegg WRL et al. (2017) A multi-species synthesis of physiological mechanisms in drought-induced tree mortality. Nat Ecol Evol 1:1285–1291. 29046541 10.1038/s41559-017-0248-x

[ref2] Allen CD, Breshears DD, McDowell NG (2015) On underestimation of global vulnerability to tree mortality and forest die-off from hotter drought in the Anthropocene. Ecosphere 6:1–55.

[ref3] Anderegg WRL, Klein T, Bartlett M, Sack L, Pellegrini AFA, Choat B, Jansen S (2016) Meta-analysis reveals that hydraulic traits explain cross-species patterns of drought-induced tree mortality across the globe. Proc Natl Acad Sci USA 113:5024–5029. 27091965 10.1073/pnas.1525678113 PMC4983847

[ref4] Aspinwall MJ, Loik ME, Resco de Dios V, Tjoelker MG, Payton PR, Tissue DT (2015) Utilizing intraspecific variation in phenotypic plasticity to bolster agricultural and forest productivity under climate change. Plant Cell Environ 38:1752–1764. 25132508 10.1111/pce.12424

[ref5] Bartlett MK, Scoffoni C, Sack L (2012) The determinants of leaf turgor loss point and prediction of drought tolerance of species and biomes: a global meta-analysis. Ecol Lett 15:393–405. 22435987 10.1111/j.1461-0248.2012.01751.x

[ref6] Bartlett MK, Zhang Y, Kreidler N, Sun SW, Ardy R, Cao KF, Sack L (2014) Global analysis of plasticity in turgor loss point, a key drought tolerance trait. Ecol Lett 17:1580–1590. 25327976 10.1111/ele.12374

[ref7] Billon LM, Blackman CJ, Cochard H, Badel E, Hitmi A, Cartailler J, Souchal R, Torres-Ruiz JM (2020) The DroughtBox: a new tool for phenotyping residual branch conductance and its temperature dependence during drought. Plant Cell Environ 43:1584–1594. 32187686 10.1111/pce.13750

[ref10] Blackman CJ, Pfautsch S, Choat B, Delzon S, Gleason SM, Duursma RA (2016) Toward an index of desiccation time to tree mortality under drought. Plant Cell Environ 39:2342–2345. 27093688 10.1111/pce.12758

[ref8] Blackman CJ, Creek D, Maier C et al. (2019 *a*) Drought response strategies and hydraulic traits contribute to mechanistic understanding of plant dry-down to hydraulic failure. Tree Physiol 39:910–924. 30865274 10.1093/treephys/tpz016

[ref9] Blackman CJ, Li X, Choat B, Rymer PD, De Kauwe MG, Duursma RA, Tissue DT, Medlyn BE (2019 *b*) Desiccation time during drought is highly predictable across species of *Eucalyptus* from contrasting climates. New Phytol 224:632–643. 31264226 10.1111/nph.16042

[ref11] Britton TG, Brodribb TJ, Richards SA, Ridley C, Hovenden MJ (2022) Canopy damage during a natural drought depends on species identity, physiology and stand composition. New Phytol 233:2058–2070. 34850394 10.1111/nph.17888

[ref12] Brodribb TJ, Powers J, Cochard H, Choat B (2020) Hanging by a thread? Forests and drought. Science 368:261–266. 32299945 10.1126/science.aat7631

[ref13] Buckley TN (2019) How do stomata respond to water status? New Phytol 224:21–36. 31069803 10.1111/nph.15899

[ref14] Bueno A, Alfarhan A, Arand K et al. (2019) Effects of temperature on the cuticular transpiration barrier of two desert plants with water-spender and water-saver strategies. J Exp Bot 70:1613–1625. 30715440 10.1093/jxb/erz018 PMC6416792

[ref15] Bueno A, Sancho-Knapik D, Gil-Pelegrín E, Leide J, Peguero-Pina JJ, Burghardt M, Riederer M (2020) Cuticular wax coverage and its transpiration barrier properties in *Quercus coccifera* L. leaves: Does the environment matter? Tree Physiol 40:827–840. 31728539 10.1093/treephys/tpz110

[ref16] Chitra-Tarak R, Xu C, Aguilar S et al. (2021) Hydraulically-vulnerable trees survive on deep-water access during droughts in a tropical forest. New Phytol 231:1798–1813. 33993520 10.1111/nph.17464 PMC8457149

[ref17] Choat B, Brodribb TJ, Brodersen CR, Duursma RA, Lopez R, Medlyn BE (2018) Triggers of tree mortality under drought. Nature 558:531–539. 29950621 10.1038/s41586-018-0240-x

[ref18] Cochard H, Damour G, Bodet C, Tharwat I, Poirier M, Ameglio T (2005) Evaluation of a new centrifuge technique for rapid generation of xylem vulnerability curves. Physiol Plant 124:410–418.

[ref18a] Cochard, H (2006) Cavitation in trees. Comptes Rendus Physique 7:1018–1026.

[ref19] Cochard H, Pimont F, Ruffault J, Martin-StPaul N (2021) SurEau: a mechanistic model of plant water relations under extreme drought. Ann For Sci 78:1–23.

[ref20] Creek D, Lamarque LJ, Torres-Ruiz JM, Parise C, Burlett R, Tissue DT, Delzon S (2020) Xylem embolism in leaves does not occur with open stomata: evidence from direct observations using the optical visualization technique. J Exp Bot 71:1151–1159. 31641746 10.1093/jxb/erz474

[ref21] Davis SD, Ewers F, Sperry J, Portwood KA, Crocker MC, Adams GC (2002) Shoot dieback during prolonged drought in *Ceanothus* (Rhamnaceae) chaparral of California: a possible case of hydraulic failure. Am J Bot 89:820–828. 21665682 10.3732/ajb.89.5.820

[ref22] De Kauwe MG, Medlyn BE, Ukkola AM et al. (2020) Identifying areas at risk of drought-induced tree mortality across South-Eastern Australia. Glob Chang Biol 26:5716–5733. 32512628 10.1111/gcb.15215

[ref23] Duursma RA, Blackman CJ, Lopéz R, Martin-StPaul NK, Cochard H, Medlyn BE (2019) On the minimum leaf conductance: its role in models of plant water use, and ecological and environmental controls. New Phytol 221:693–705. 30144393 10.1111/nph.15395

[ref24] Fensham RJ, Fairfax RJ (2007) Drought-related tree death of savanna eucalypts: species susceptibility, soil conditions and root architecture. J Veg Sci 18:71–80.

[ref25] Gleason SM, Blackman CJ, Cook AM, Laws CA, Westoby M (2014) Whole-plant capacitance, embolism resistance and slow transpiration rates all contribute to longer desiccation times in woody angiosperms from arid and wet habitats. Tree Physiol 34:275–284. 24550089 10.1093/treephys/tpu001

[ref26] Hammond WM, Williams AP, Abatzoglou JT et al. (2022) Global field observations of tree die-off reveal hotter-drought fingerprint for Earth’s forests. Nat Commun 13:1–11. 35383157 10.1038/s41467-022-29289-2 PMC8983702

[ref27] Holtta T, Mencuccini M, Nikinmaa E (2011) A carbon cost-gain model explains the observed patterns of xylem safety and efficiency. Plant Cell Environ 34:1819–1834. 21689111 10.1111/j.1365-3040.2011.02377.x

[ref28] IPCC (2022) Climate change 2022: impacts, adaptation, and vulnerability. Contribution of Working Group II to the sixth assessment report of the Intergovernmental Panel on Climate Change. Cambridge University Press, UK and New York, NY, USA.

[ref29] Jacobsen AL, Pratt RB (2018) Extensive drought-associated plant mortality as an agent of type-conversion in chaparral shrublands. New Phytol 219:498–504. 29727471 10.1111/nph.15186

[ref30] Klein T, Torres-Ruiz JM, Albers JJ (2022) Conifer desiccation in the 2021 NW heatwave confirms the role of hydraulic damage. Tree Physiol 42:722–726. 35084498 10.1093/treephys/tpac007

[ref31] Koçillari L, Olson ME, Suweis S et al. (2021) The widened pipe model of plant hydraulic evolution. Proc Natl Acad Sci USA 118:e2100314118. 34039710 10.1073/pnas.2100314118 PMC8179198

[ref32] Lamarque LJ, Delzon S, Toups H et al. (2020) Over-accumulation of abscisic acid in transgenic tomato plants increases the risk of hydraulic failure. Plant Cell Environ 43:548–562. 31850535 10.1111/pce.13703

[ref33] Lamy JB, Delzon S, Bouche PS, Alia R, Vendramin GG, Cochard H, Plomion C (2014) Limited genetic variability and phenotypic plasticity detected for cavitation resistance in a Mediterranean pine. New Phytol 201:874–886. 24180459 10.1111/nph.12556

[ref34] Lemaire C, Blackman CJ, Cochard H, Menezes-Silva PE, Torres-Ruiz JM, Herbette S (2021) Acclimation of hydraulic and morphological traits to water deficit delays hydraulic failure during simulated drought in poplar. Tree Physiol 41:2008–2021. 34259313 10.1093/treephys/tpab086

[ref34a] Lobo A, Torres-Ruiz JM, Burlett R, Lemaire C, Parise C, Francioni C, Truffaut L, Tomaskova I, Hansen JK, Kjaer ED, Kremer A and Delzon S (2018) Assessing inter- and intraspecific variability of xylem vulnerability to embolism in oaks. Forest Ecology and Management 424:53–61. 29910530 10.1016/j.foreco.2018.04.031 PMC5997172

[ref35] Lopez R, Cano FJ, Choat B, Cochard H, Gil L (2016) Plasticity in vulnerability to cavitation of *Pinus canariensis* occurs only at the driest end of an aridity gradient. Front Plant Sci 7:1–10. 27375637 10.3389/fpls.2016.00769 PMC4891331

[ref36] López R, Cano FJ, Martin-StPaul NK, Cochard H, Choat B (2021) Coordination of stem and leaf traits define different strategies to regulate water loss and tolerance ranges to aridity. New Phytol 230:497–509. 33452823 10.1111/nph.17185

[ref37] Loram-Lourenço L, Farnese FS, Alves RDFB et al. (2022) Variations in bark structural properties affect both water loss and carbon economics in neotropical savanna trees in the Cerrado region of Brazil. J Ecol 110:1826–1843.

[ref38] Machado R, Loram-Lourenço L, Farnese FS et al. (2021) Where do leaf water leaks come from? Trade-offs underlying the variability in minimum conductance across tropical savanna species with contrasting growth strategies. New Phytol 229:1415–1430. 32964437 10.1111/nph.16941

[ref39] Mantova M, Herbette S, Cochard H, Torres-Ruiz JM (2022) Hydraulic failure and tree mortality: from correlation to causation. Trends Plant Sci 27:335–345. 10.1016/j.tplants.2021.10.003. 34772610

[ref40] Martin-StPaul N, Delzon S, Cochard H (2017) Plant resistance to drought depends on timely stomatal closure. Ecol Lett 20:1437–1447. 28922708 10.1111/ele.12851

[ref41] McDowell NG, Sapes G, Pivovaroff A et al. (2022) Mechanisms of woody-plant mortality under rising drought, CO2 and vapour pressure deficit. Nat Rev Earth Environ 3:294–308.

[ref42] Mencuccini M, Bonosi L (2001) Leaf/sapwood area ratios in Scots pine show acclimation across Europe. Can J For Res 31:442–456.

[ref43] Mencuccini M, Manzoni S, Christoffersen B (2019) Modelling water fluxes in plants: from tissues to biosphere. New Phytol 222:1207–1222. 30636295 10.1111/nph.15681

[ref44] Mendez-Alonzo R, Paz H, Zuluaga RC, Rosell JA, Olson ME (2012) Coordinated evolution of leaf and stem economics in tropical dry forest trees. Ecology 93:2397–2406. 23236911 10.1890/11-1213.1

[ref45] Mitchell PJ, O'Grady AP (2015) Adaptation of leaf water relations to climatic and habitat water availability. Forests 6:2281–2295.

[ref46] Nardini A, Casolo V, Dal Borgo A, Savi T, Stenni B, Bertoncin P, Zini L, McDowell NG (2016) Rooting depth, water relations and non-structural carbohydrate dynamics in three woody angiosperms differentially affected by an extreme summer drought. Plant Cell Environ 39:618–627. 26437327 10.1111/pce.12646

[ref47] Nolan RH, Gauthey A, Losso A et al. (2021) Hydraulic failure and tree size linked with canopy die-back in eucalypt forest during extreme drought. New Phytol 230:1354–1365. 33629360 10.1111/nph.17298

[ref48] Paddock WA III, Davis SD, Pratt RB, Jacobsen AL, Tobin MF, López-Portillo J, Ewers FW (2013) Factors determining mortality of adult chaparral shrubs in an extreme drought year in California. Aliso 31:49–57.

[ref49] Pineda-Garcia F, Paz H, Meinzer FC (2013) Drought resistance in early and late secondary successional species from a tropical dry forest: the interplay between xylem resistance to embolism, sapwood water storage and leaf shedding. Plant Cell Environ 36:405–418. 22812458 10.1111/j.1365-3040.2012.02582.x

[ref51] Pivovaroff AL, Pasquini SC, De Guzman ME, Alstad KP, Stemke JS, Santiago LS (2016) Multiple strategies for drought survival among woody plant species. Funct Ecol 30:517–526.

[ref50] Pivovaroff AL, McDowell NG, Rodrigues TB et al. (2021) Stability of tropical forest tree carbon-water relations in a rainfall exclusion treatment through shifts in effective water uptake depth. Glob Chang Biol 27:6454–6466. 34469040 10.1111/gcb.15869

[ref52] Pratt RB, Jacobsen AL, Ewers FW, Davis SD (2007) Relationships among xylem transport, biomechanics and storage in stems and roots of nine Rhamnaceae species of the California chaparral. New Phytol 174:787–798. 17504462 10.1111/j.1469-8137.2007.02061.x

[ref53] Ribeiro DR, Silva JLA, do Nascimento MT, Vitória AP (2021) Leaf habits and their relationship with leaf and wood traits in tropical dry forests. Trees 36:7–24.

[ref54] Ripullone F, Camarero JJ, Colangelo M, Voltas J (2020) Variation in the access to deep soil water pools explains tree-to-tree differences in drought-triggered dieback of Mediterranean oaks. Tree Physiol 40:591–604. 32159804 10.1093/treephys/tpaa026

[ref56] Ruffault J, Pimont F, Cochard H, Dupuy J-L, Martin-StPaul NK (2022) SurEau-Ecos v2. 0: a trait-based plant hydraulics model for simulations of plant water status and drought-induced mortality at the ecosystem level. Geosci Model Dev 15:5593–5626.

[ref55] Ruffault J, Limousin JM, Pimont F et al. (2023) Plant hydraulic modelling of leaf and canopy fuel moisture content reveals increasing vulnerability of a Mediterranean forest to wildfires under extreme drought. New Phytol 237:1256–1269. 36366950 10.1111/nph.18614

[ref57] Scholz FG, Phillips N, Bucci SJ, Meinzer FC, Goldstein G (2011) Hydraulic capacitance: biophysics and functional significance of internal water sources in relation to tree size. In: Meinzer FC, Lachenbruch B, Dawson TE (eds) Size- and age-related changes in tree structure and function. Springer, Dordrecht, pp 341–361.

[ref58] Schuldt B, Knutzen F, Delzon S, Jansen S, Müller-Haubold H, Burlett R, Clough Y, Leuschner C (2016) How adaptable is the hydraulic system of European beech in the face of climate change-related precipitation reduction? New Phytol 210:443–458. 26720626 10.1111/nph.13798

[ref59] Schuster AC, Burghardt M, Riederer M (2017) The ecophysiology of leaf cuticular transpiration: are cuticular water permeabilities adapted to ecological conditions? J Exp Bot 68:5271–5279. 29036342 10.1093/jxb/erx321

[ref60] Skelton RP, Dawson TE, Thompson SE, Shen Y, Weitz AP, Ackerly D (2018) Low vulnerability to xylem embolism in leaves and stems of North American oaks. Plant Physiol 177:1066–1077. 29789436 10.1104/pp.18.00103 PMC6052988

[ref61] Sobol IM (2001) Global sensitivity indices for nonlinear mathematical models and their Monte Carlo estimates. Math Comput Simul 55:271–280.

[ref62] Sperry JS, Love DM (2015) What plant hydraulics can tell us about responses to climate-change droughts. New Phytol 207:14–27. 25773898 10.1111/nph.13354

[ref63] Sperry JS, Venturas MD, Anderegg WR, Mencuccini M, Mackay DS, Wang Y, Love DM (2017) Predicting stomatal responses to the environment from the optimization of photosynthetic gain and hydraulic cost. Plant Cell Environ 40:816–830. 27764894 10.1111/pce.12852

[ref65] Torres-Ruiz JM, Jansen S, Choat B et al. (2015) Direct X-ray microtomography observation confirms the induction of embolism upon xylem cutting under tension. Plant Physiol 167:40–43. 25378693 10.1104/pp.114.249706 PMC4281005

[ref64] Torres-Ruiz JM, Cochard H, Mencuccini M, Delzon S, Badel E (2016) Direct observation and modelling of embolism spread between xylem conduits: a case study in Scots pine. Plant Cell Environ 39:2774–2785. 27739597 10.1111/pce.12840

[ref66] Torres-Ruiz JM, Kremer A, Carins Murphy MR, Brodribb T, Lamarque LJ, Truffaut L, Bonne F, Ducousso A, Delzon S (2019) Genetic differentiation in functional traits among European sessile oak populations. Tree Physiol 39:1736–1749. 31553461 10.1093/treephys/tpz090 PMC6954098

[ref68] Tyree MT, Ewers FW (1991) The hydraulic architecture of trees and other woody-plants. New Phytol 119:345–360.

[ref69] Tyree MT, Hammel HT (1972) Measurement of turgor pressure and water relations of plants by pressure-bomb technique. J Exp Bot 23:267–282.

[ref70] Tyree MT, Sperry JS (1989) Vulnerability of xylem to cavitation and embolism. Annu Rev Plant Physiol Plant Mol Biol 40:19–38.

[ref67] Tyree MT, Cochard H, Cruiziat P, Sinclair B, Ameglio T (1993) Drought-induced leaf shedding in walnut: evidence for vulnerability segmentation. Plant Cell Environ 16:879–882.

[ref71] Venturas MD, MacKinnon ED, Dario HL, Jacobsen AL, Pratt RB, Davis SD (2016) Chaparral shrub hydraulic traits, size, and life history types relate to species mortality during California’s historic drought of 2014. PLoS One 11:e0159145. 10.1371/journal.pone.0159145. 27391489 PMC4938587

[ref72] Venturas MD, Sperry JS, Love DM, Frehner EH, Allred MG, Wang Y, Anderegg WR (2018) A stomatal control model based on optimization of carbon gain versus hydraulic risk predicts aspen sapling responses to drought. New Phytol 220:836–850. 29998567 10.1111/nph.15333

[ref73] Wolfe BT (2017) Retention of stored water enables tropical tree saplings to survive extreme drought conditions. Tree Physiol 37:469–480. 28338739 10.1093/treephys/tpx001

[ref74] Wolfe BT (2020) Bark water vapour conductance is associated with drought performance in tropical trees. Biol Lett 16:20200263. 32750268 10.1098/rsbl.2020.0263 PMC7480154

[ref75] Wolfe BT, Sperry JS, Kursar TA (2016) Does leaf shedding protect stems from cavitation during seasonal droughts? A test of the hydraulic fuse hypothesis. New Phytol 212:1007–1018. 27373446 10.1111/nph.14087

[ref76] Zhu SD, Liu H, Xu QY, Cao KF, Ye Q (2016) Are leaves more vulnerable to cavitation than branches? Funct Ecol 30:1740–1744.

