# Supplementary material for: Key hydraulic traits control the dynamics of plant dehydration in four contrasting tree species during drought
Source: Tree Physiol. 2023 Jun 15;43(10):1772–83. doi: 10.1093/treephys/tpad075 (PMC10652334; doi:10.1093/treephys/tpad075)
Supplement: Supporting_Information_Fig_S1_tpad075 [file supporting_information_fig_s1_tpad075.docx]

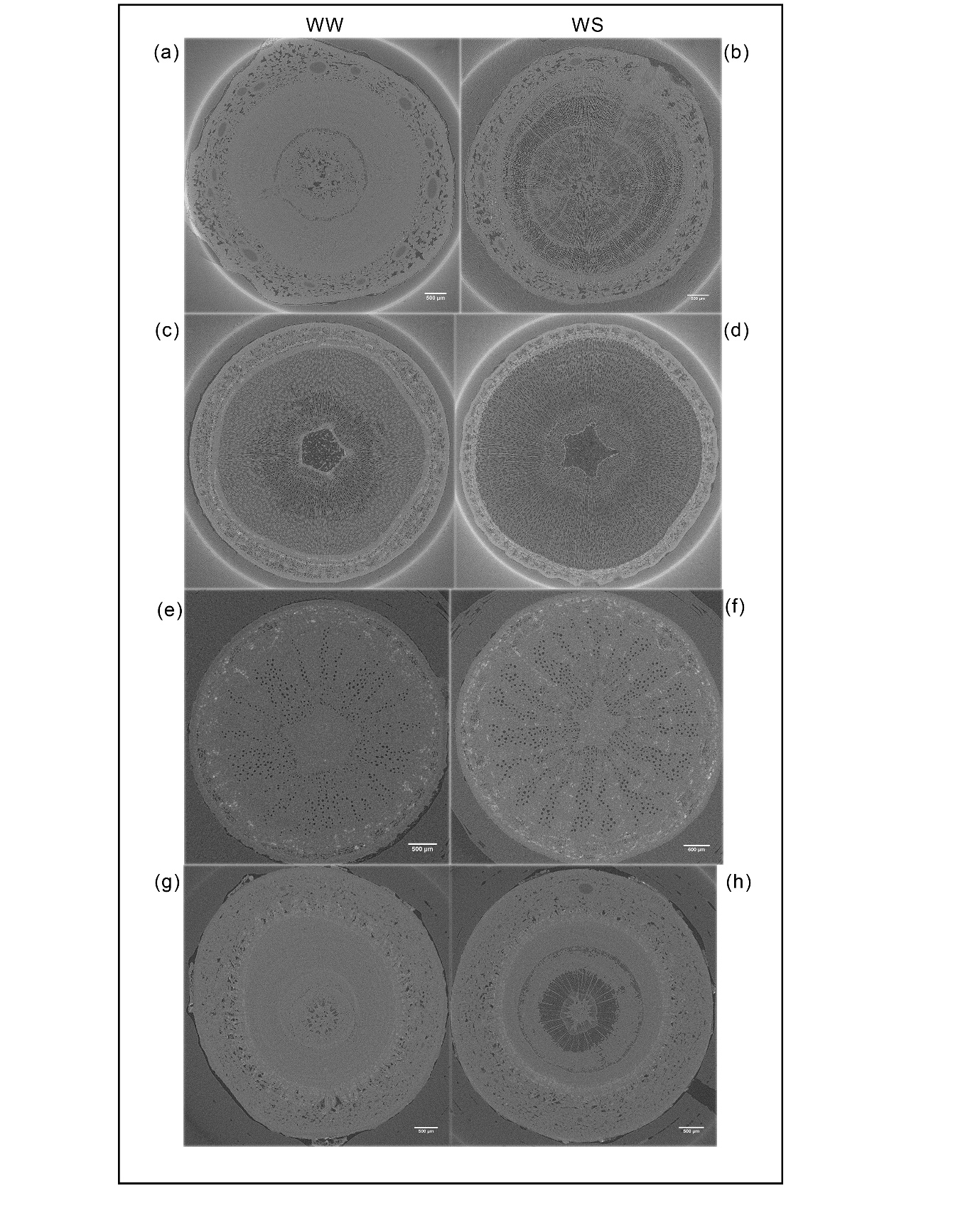


Supporting Information Fig. S1. Representative micro-CT images of control (WW) and droughted (WS) branch samples taken from individuals of each of the four species (plots a,b = *Pinus halepensis*; plots c,d = *Populus nigra*; plots e,f = *Quercus ilex*; and plots g,h = *Cedrus atlantica*) shortly after rewatering from peak drought. These images were used to infer percentage loss of conductance (PLC) due to embolism formatting during the experiment. In the images of WS *Pinus halepensis*, *Populus nigra*, and *C. atlantica*, drought induced embolisms are represented by open (dark) conduits within the xylem. For *Q. ilex*, open vessels were present in both WS and WW individuals, most likely because of sampling artefacts.
