# Supplementary material for: Key hydraulic traits control the dynamics of plant dehydration in four contrasting tree species during drought
Source: Tree Physiol. 2023 Jun 15;43(10):1772–83. doi: 10.1093/treephys/tpad075 (PMC10652334; doi:10.1093/treephys/tpad075)
Supplement: Supporting_Information_Fig_S2_tpad075 [file supporting_information_fig_s2_tpad075.docx]

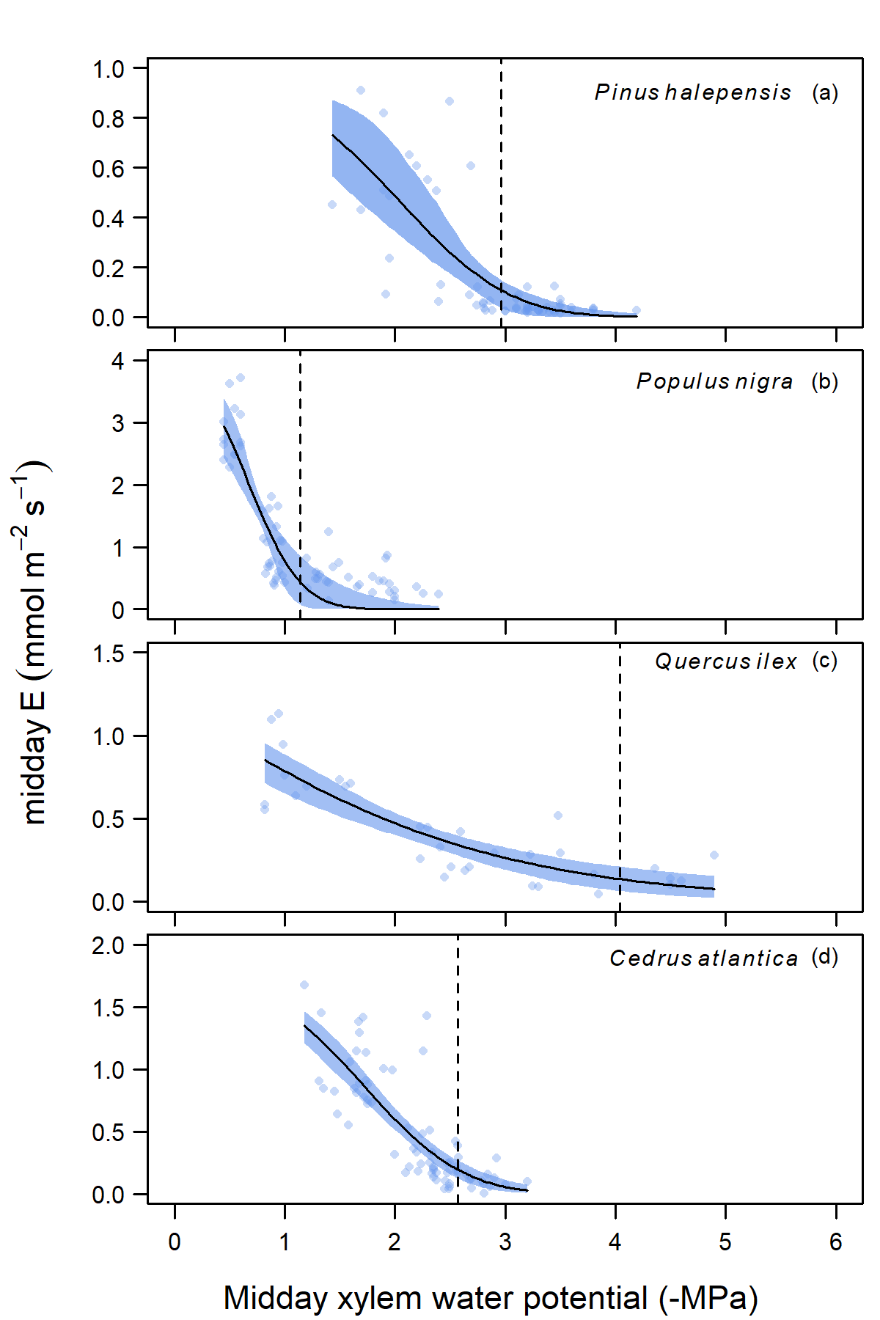


Supporting Information Fig. S2. The relationship between midday transpiration (*E*) and decreasing midday water potential (-MPa) in plants of *Pinus halepensis* (a), *Populus nigra* (b), *Quercus ilex* (c), and *Cedrus atlantica* (d) during dry-down. In each plot, the data are fitted with a ‘Weibull’ model using the *fitplc* package. Vertical dashed lines indicate the water potential at 88% reduction in midday *E*. This value was used to represent stomatal closure (*P_gs_*_88_) in the model.
