# Supplementary material for: Key hydraulic traits control the dynamics of plant dehydration in four contrasting tree species during drought
Source: Tree Physiol. 2023 Jun 15;43(10):1772–83. doi: 10.1093/treephys/tpad075 (PMC10652334; doi:10.1093/treephys/tpad075)
Supplement: Supporting_Information_Fig_S3_tpad075 [file supporting_information_fig_s3_tpad075.docx]

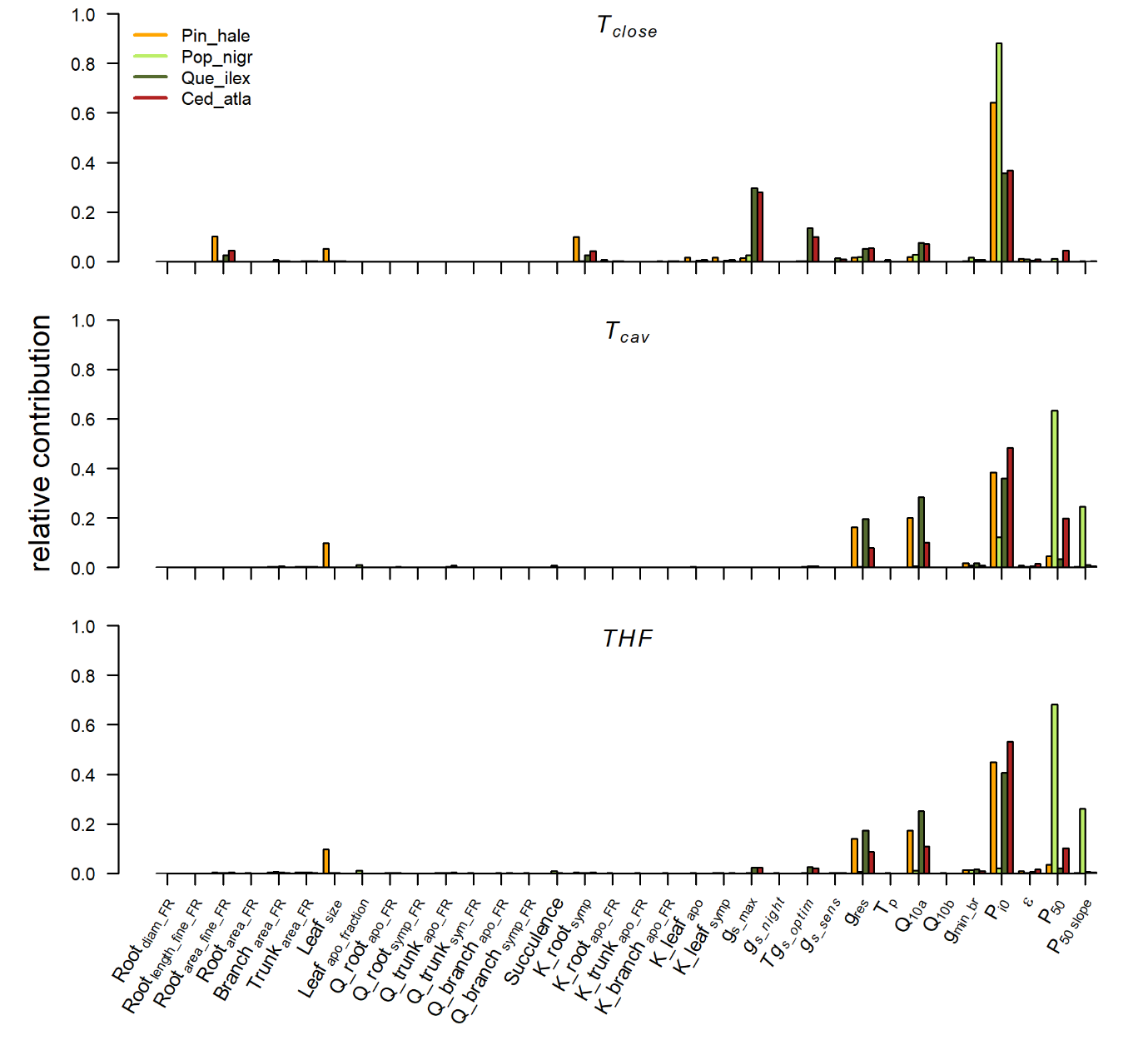


Supporting Information Fig. S3 Global sensitivity analysis, focusing on 34 representative hydraulic and allometric traits and their relative contribution (i.e., their Sobol’s total order indices) to determining dehydration times from full hydration to stomatal closure (Tclose), the subsequent time to hydraulic failure (Tcav), and the overall time from full hydration to hydraulic failure (THF). Traits beginning with ‘Q_’ represent organ specific apoplasmic (apo) and symplasmic (symp) water content; traits beginning with ‘K_’ represent organ specific conductance. Traits ending with ‘FR’ were calculated via a representative ‘fractal tree’ (see Cochard et al. 2021). Colours represent the four species in the study: Pin_hale = Pinus halepnsis (orange); Pop_nigr = Populus nigra (light green); Que_ilex = Quercus ilex (dark green); Ced_atla = Cedrus atlantica (red). See Table 1 and Table S1 for full trait details and Table S3 for global sensitivity analysis output of individual species traits.
