# Supplementary material for: Key hydraulic traits control the dynamics of plant dehydration in four contrasting tree species during drought
Source: Tree Physiol. 2023 Jun 15;43(10):1772–83. doi: 10.1093/treephys/tpad075 (PMC10652334; doi:10.1093/treephys/tpad075)
Supplement: Supporting_Information_Fig_S4_tpad075 [file supporting_information_fig_s4_tpad075.docx]

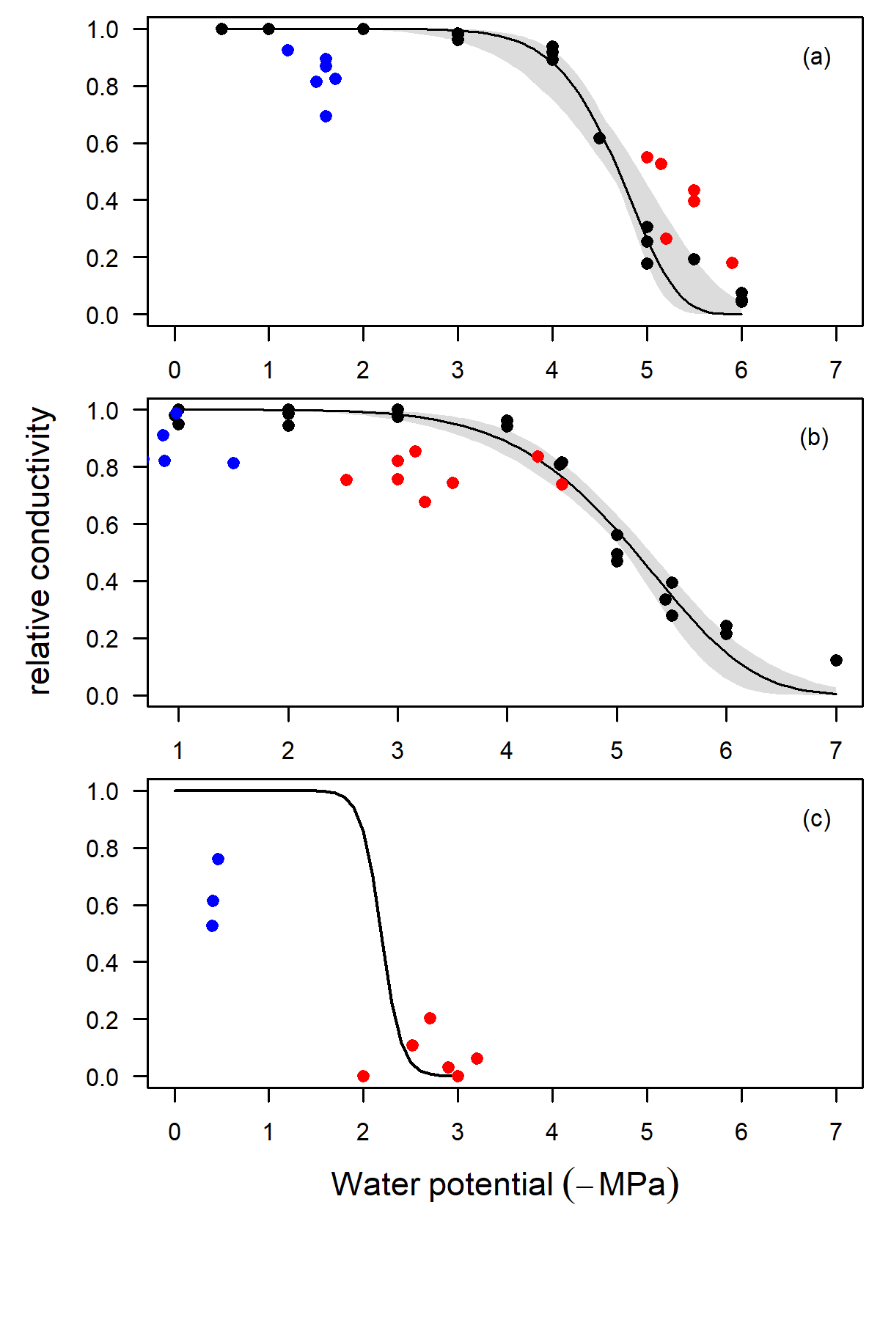


Supporting Information Fig. S4. Plots showing good correspondence between levels of embolism expressed in terms of relative conductivity recorded immediately following peak drought in well-watered controls (blue) and water stressed plants (red) relative to each species vulnerability curve. Plot (a) = *Pinus halepensis*, plot (b) = *Cedrus atlantica*, and plot (c) = *Populus nigra*. Levels of embolism in experimental plants were determined using micro-CT. Vulnerability curves were either sourced from the literature (*Pinus halepensis* and *C. atlantica* (Cochard et al 2006)) or from unpublished data (*Populus nigra* (Cochard unpublished)).
