# Supplementary material for: Key hydraulic traits control the dynamics of plant dehydration in four contrasting tree species during drought
Source: Tree Physiol. 2023 Jun 15;43(10):1772–83. doi: 10.1093/treephys/tpad075 (PMC10652334; doi:10.1093/treephys/tpad075)
Supplement: Supporting_Information_Fig_S5_tpad075 [file supporting_information_fig_s5_tpad075.docx]

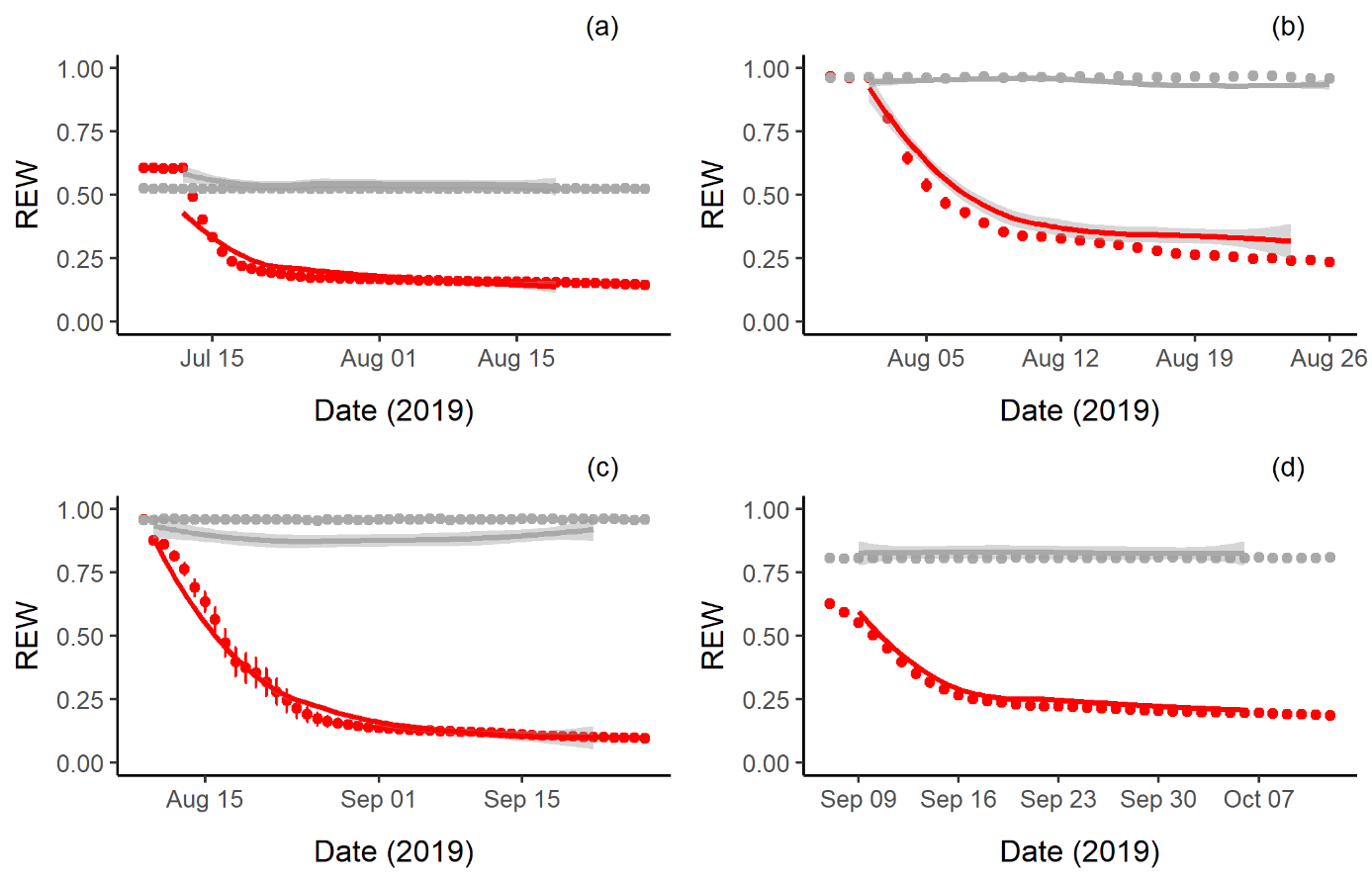


Supporting Information Fig. S5. Plots showing strong correspondence between observed (circles, plus 95% confidence interval error bars) and modelled (lines, plus 95% confidence intervals represented by grey shading) changes in relative extractable soil water content during drought in WS (red) and WW (grey) plants of the four study species: (a) *Pinus halepensis*; (b) *Populus nigra*; (c) *Quercus ilex*; (d) *Cedrus atlantica*. We used changes in relative extractable soil water content as a measure of changes in plant transpiration.
