# Supplementary material for: Key hydraulic traits control the dynamics of plant dehydration in four contrasting tree species during drought
Source: Tree Physiol. 2023 Jun 15;43(10):1772–83. doi: 10.1093/treephys/tpad075 (PMC10652334; doi:10.1093/treephys/tpad075)
Supplement: Supporting_Information_Fig_S6_tpad075 [file supporting_information_fig_s6_tpad075.docx]

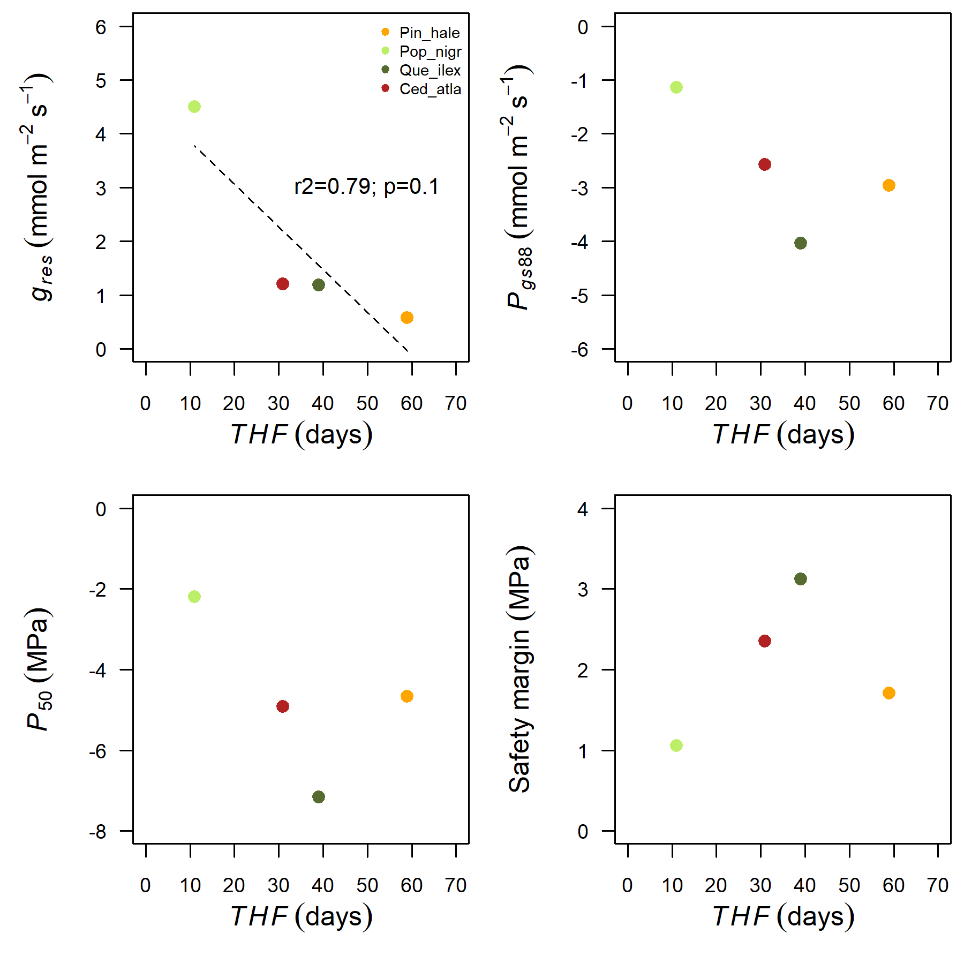


Supporting Information Fig. S6. Plots showing a lack of strong correlation across the four study species between selected plant traits commonly linked to drought tolerance and modelled time to hydraulic failure.
